# A Rare Case of Duplicated Gallbladder in the United Arab Emirates

**DOI:** 10.7759/cureus.57061

**Published:** 2024-03-27

**Authors:** Raya Zahid Ahmad, Sarah Fadhil Hussein Ghayb Al-Rubaye, Reema Fathima, Fatima Buti, Hajer Busharar

**Affiliations:** 1 Surgery, Dubai Medical University, Dubai, ARE; 2 Surgery, Dubai Academic Health Corporation, Dubai, ARE; 3 General Surgery, Rashid Hospital, Dubai Health, Dubai, ARE

**Keywords:** gallbladder malformations, septate gallbladder duplication, laparoscopic cholecystectomy, cholelithiasis, chronic cholecystitis

## Abstract

Gallbladder duplication is a rare congenital aberration that requires special attention due to its clinical, diagnostic, and surgical complexity. Its symptoms are usually consistent with cholecystitis and other gallbladder etiologies. This is a case report of a 39-year-old male patient with a known case of chronic cholecystitis and cholelithiasis. He presented with mild epigastric abdominal pain over two months; as a result, he opted for elective cholecystectomy. Subsequently, a type I septate duplicated gallbladder was incidentally diagnosed following a histopathology report. This literature is the first report of a case of septate gallbladder duplication presenting with cholecystitis in the United Arab Emirates.

## Introduction

Gallbladder duplication is a rare congenital variation with an incidence of 1:4000 [1,2]. It has no predisposing factors like age or ethnicity; however, some data suggest a twice higher incidence in women than men [1,2]. It is commonly encountered during surgical procedures and autopsies [1,2]. Patients with a duplicated gallbladder are either asymptomatic or present with symptoms correlating with cholecystitis and cholelithiasis, such as epigastric postprandial abdominal pain, nausea, vomiting, and anorexia [2,3]. The duplicated gallbladder can be classified into type I, having a single cystic duct, and type II, having separate cystic ducts for each gallbladder [4,5]. The patient, in this case, had a type I septate gallbladder. Among different diagnostic modalities usually performed on a surgical patient, ultrasound, and computed tomography (CT) may not give an apt visualization of the hepatic region to identify such anatomic anomalies [4,6]. Hence, magnetic resonance cholangiopancreatography (MRCP) and endoscopic retrograde cholangiopancreatography (ERCP) are instead considered the gold standard for diagnostic purposes [2,4].

This case report was presented twice as a poster. Once at Dubai Medical University’s first annual Research Day on April 27, 2023, and another time at the Emirates Surgical Pathology Conference on December 16, 2023.

## Case presentation

A 39-year-old male who was a known case of chronic cholelithiasis presented to the general surgery outpatient department with a two-month history of mild epigastric abdominal pain that is non-radiating and associated with mild nausea.

On examination, the patient was alert, stable, and afebrile, but mild tenderness was present over the right upper quadrant with a positive Murphy sign. The patient’s BMI was 33.69 kg/m^2^, falling under the obese category.

His lab work, including his complete blood count, urea, electrolytes, liver enzymes, liver function, and kidney function test, revealed no abnormal findings or significant changes in their values.

**Figure 1:**
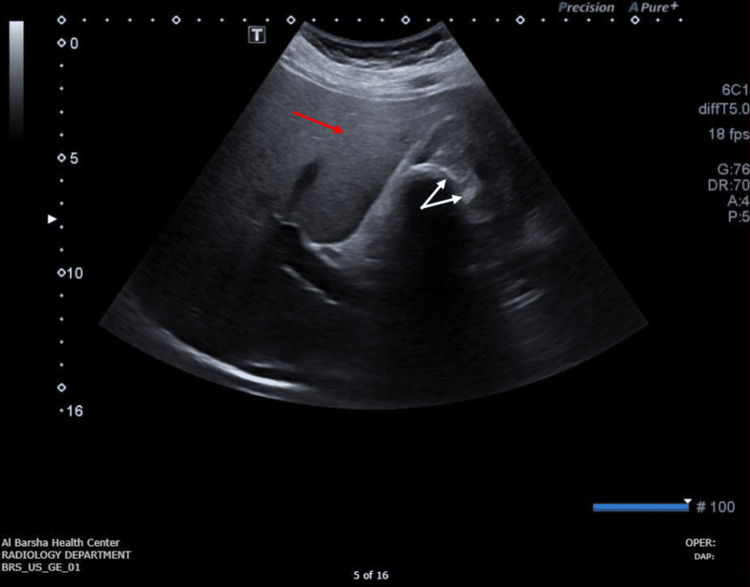
Abdominal ultrasound showing a few intraluminal gallbladder calculi, the largest measuring 2.1 X 2.5 cm with intraluminal debris and mild diffuse wall thickening represented by the white arrows. The liver is mildly enlarged (17 cm), with a bright echo pattern, smooth, regular surface, no focal lesion, and no dilated intrahepatic biliary radicals - normal hepatic veins represented by the red arrow Red arrow: liver; white arrows: gallbladder calculi Not mentioned in the figure but noted while performing the ultrasound: areas of focal fat sparing are noted within the right lobe. The portal vein is not dilated and shows normal hepatorenal flow. There is no abnormal vascularity and no pericholecystic collection. Common bile duct (CBD): This is prominent at porta (6 mm); however, the distal CBD is obscured by bowel gasses. The pancreas, spleen, and kidneys are of standard size with no abnormalities.

On May 5, 2021, an ultrasound (USS) of the abdomen was done, and its features suggest chronic calculus cholecystitis and grade one fatty infiltration of the liver with mild hepatomegaly, as seen in Figure 1.

He was advised for laparoscopic cholecystectomy and was scheduled on June 24, 2021, and admitted the day prior.

During the procedure, a 10 mm incision was made supraumbilical, and pneumoperitoneum was established via the Veress needle. Then, the 10 mm trocar was inserted, introducing the scope. The intraoperative finding showed a distended and thickened gallbladder; mild adhesions were found over the calot's triangle. The calot's triangle was dissected, and the cystic artery and duct were clipped. The gallbladder was then retrieved and sent for histopathology. The patient faced no complications postoperatively and was discharged on antibiotics the next day, June 25, 2021.

**Figure 2:**
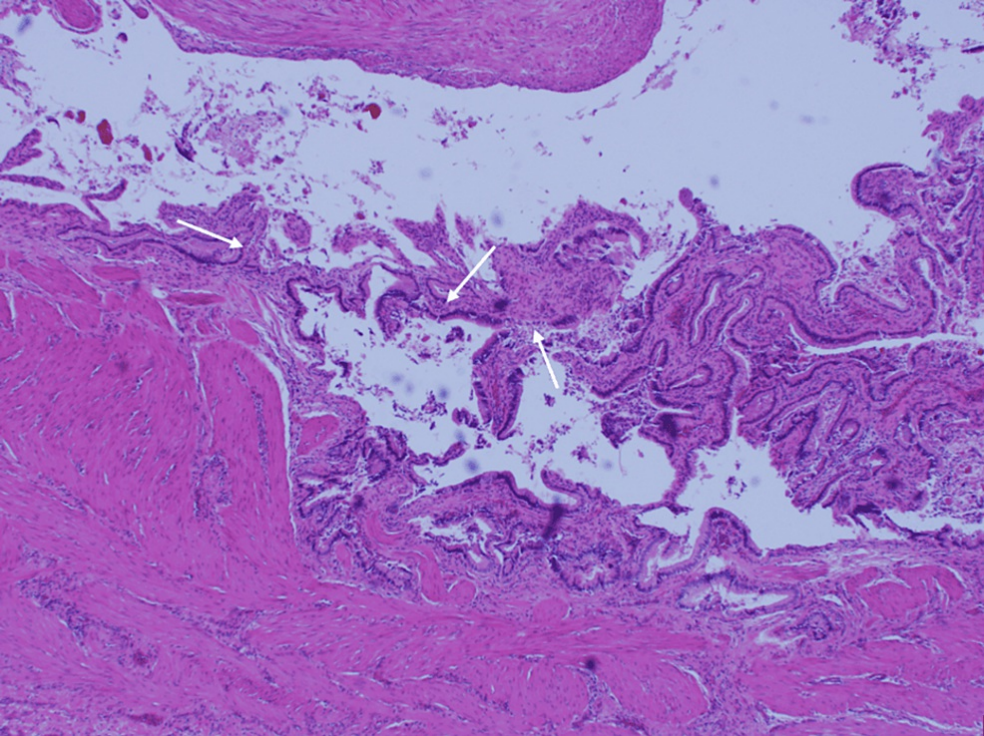
Histopathology slides of the gallbladder and its compartments showing inflammatory changes, a few of which are indicated by the white arrows Hematoxylin and eosin stain, 20x White arrows: inflammatory infiltrate

**Figure 3:**
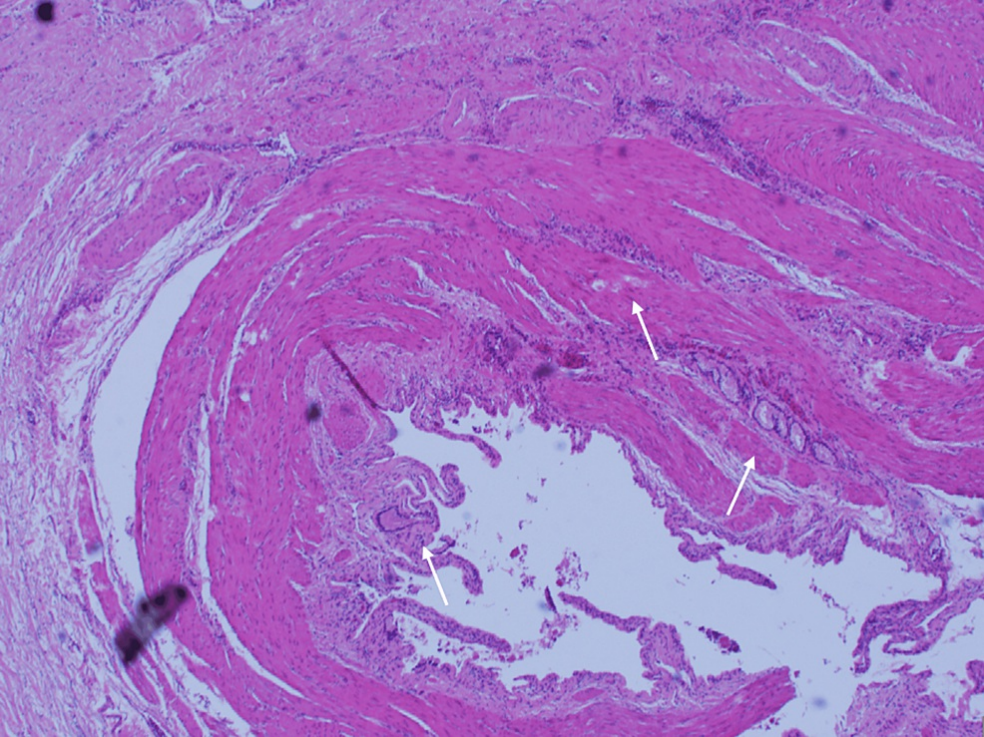
Histopathology slides of the gallbladder and its compartments showing inflammatory changes, a few of which are indicated by white arrows Hematoxylin and eosin stain, 20x White arrows: inflammatory infiltrates

Figure 2 and Figure 3 show the histopathology report. In formalin was a gallbladder measuring 8.5 x 5.5 × 2.8 cm. The outer surface showed abundant areas of hemorrhage and dark brown-colored discoloration. Slicing revealed a partially duplicated gallbladder segment with a single common cystic duct measuring 5.5 x 3.0 × 2.8 cm, with the wall thickness of both gallbladders measuring 0.3 to 0.5 cm, separated by a thin brown-colored septum measuring 0.1 cm in thickness. The inner surfaces of both gallbladders were markedly congested and showed dark brown-colored discoloration. One gallbladder showed two large black-colored hard stones, the larger one measuring 1.0 x 1.0 x 0.9 cm. The duplicated gallbladder segment showed another two large black-hard stones, the larger one measuring 1.1 x 1.0 x 0.9 cm.

The findings were consistent with type I septate gallbladder duplication, a rare anatomic malformation with focal acute-on-chronic hemorrhagic cholecystitis.

## Discussion

**Figure 4:**
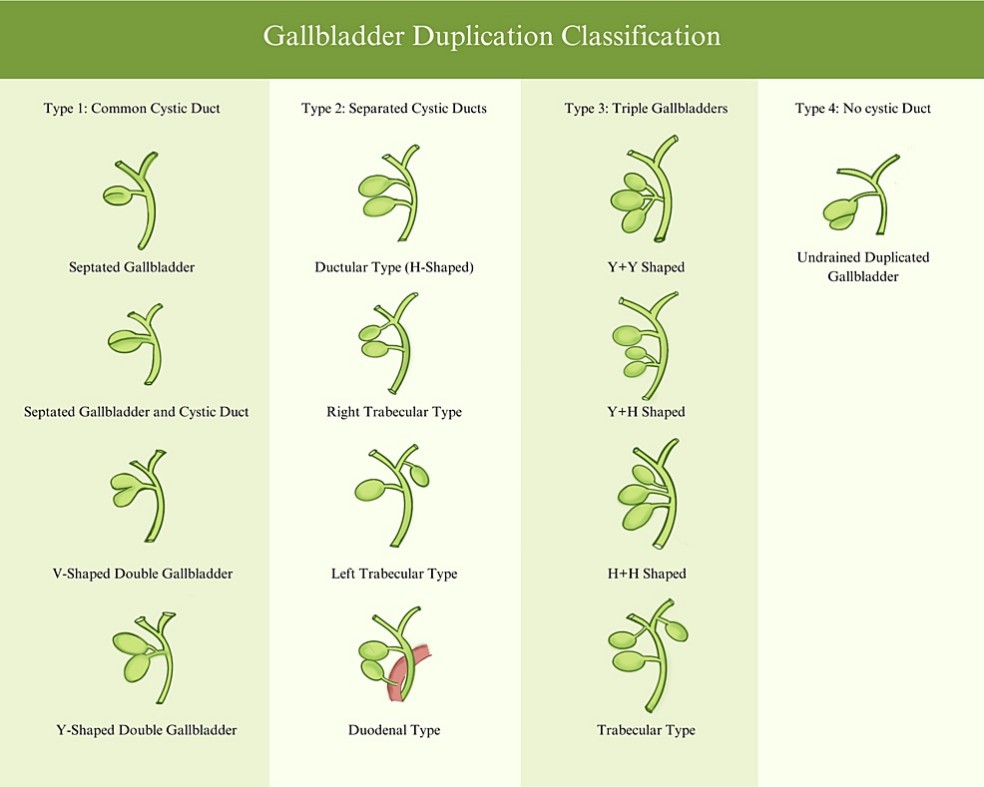
Harlafit’s gallbladder duplication types according to the time of embryological division This original work was developed by authors Raya Zahid Ahmad, Sarah Fadhil Hussein Ghayb Al-Rubaye, and Reema Fathima, and its information was extracted from Di Muzio et al. in 2023 [9] with permission from the original author and the article’s publishing website Radiopaedia.

Duplicated gallbladders come in different groups based on many factors such as the number of cystic ducts and gallbladder sacs, the draining site of the cystic ducts, and the presence of septa in the gallbladder or the cystic duct (Figure 4) [7-9]. A septate gallbladder arises due to late division (sixth week) of the primordial gallbladder sac (pars cystica), resulting in incomplete recanalization and an incomplete division with a single common cystic duct [8]. The septate gallbladder is characterized by a septum separating the gallbladder into two sections [10,11]. This type of duplication falls under the Type I split primordium group, having an incidence of 10.8%, making it one of the rarer types of anomalies [7]. This is one of the few reported cases of gallbladder duplication presenting with cholecystitis and cholelithiasis in the UAE.

Bilobed and hourglass gallbladders are the two types of septate gallbladders mentioned in the literature [10,11]. The presence of a septum dividing the gallbladder longitudinally is termed a bilobed gallbladder, whereas a septum dividing the fundus from the rest of the gallbladder transversely is termed an hourglass gallbladder [10,11]. In the latter, an opening in the septum communicates between the two compartments. The septate gallbladder can come with a single septation or multiple septations [10,11]. They can also manifest as post-inflammatory adhesions [10,11]. In the case of an hourglass gallbladder, the lower sac is more prone to bile stagnation, leading to cholelithiasis, infection, distension, and compartmentalization [10,11].

Patients with gallbladder duplication can either be asymptomatic or symptomatic [2,3]. They can present with symptoms associated with cholelithiasis, cholecystitis, cholangitis, or pancreatitis [2,3]. Hence, radiological assessment plays a crucial role in diagnosis. The initial assessment is an ultrasonographic evaluation of the gallbladder and the biliary tree due to its high sensitivity and acuity for detecting pathological findings like gallstones, cholecystitis, and anatomical variations [2,3]. Imaging differential considerations, as mentioned by Bavaresco et al. In 2022, includes gallbladder diverticulum, gallbladder fold, Phrygian cap, choledochal cyst, and focal region of pericholecystic fluid [3,7,9,12].

Further preoperative evaluation is recommended if suspicious findings are found during the ultrasound [2]. Magnetic resonance cholangiopancreatography (MRCP), oral cholecystography, tomography, and hepatobiliary iminodiacetic acid (HIDA) scan can be helpful in the structural evaluation of the biliary tree [2,3,7]. This is particularly important in gallbladder duplication. The preoperative assessments facilitate surgeons' awareness of anatomical variations, thereby reducing damage to the common bile duct, vessels, or nearby structures [2,3,7]. However, not all patients have a correct preoperative diagnosis [3,4]. Hence, surgeons should be on the lookout for anatomical variations despite normal gross morphology of the gallbladder during laparoscopic cholecystectomy [2].

Since part of the ultrasound diagnosis is skill-based, it is easy to miss an additional gallbladder without using more specific modalities. The patient’s USS did not show septations, and his liver function tests (LFT) were not deranged.

The management of duplicated gallbladder is analogous to that of other gallbladder diseases. Currently, there is no evidence of increased risk with a double gallbladder. Surgery is not indicated for incidentally discovered cases; however, if one or both gallbladders cause symptoms, cholecystectomy should be done for both gallbladders [2,4-6,8,9]. There have been reports of malignancy discovered in multiple gallbladder types; however, there seems to be no correlation between cancerous risk and congenital abnormality [8].

## Conclusions

This is a case report about a 39-year-old patient who underwent laparoscopic cholecystectomy for cholecystitis, where a septate gallbladder was incidentally discovered. Gallbladder duplication is a rare anatomical finding classified into two main types. In our case, the incidence of septate gallbladder, under type I, falls within one of the rarest types of gallbladder duplication with an incidence of only 10.8%, making this case an interesting finding. Furthermore, this is one of the few publications reporting a case of gallbladder duplication in the UAE. Missing a diagnosis of gallbladder duplication increases the risks of complications intraoperatively and postoperatively. Fortunately, our patient's surgery was successful and the gallbladder was removed, with no signs of complications and a quick discharge.
